# Chemical composition and biological investigation of *Pelargonium endlicherianum* root extracts

**DOI:** 10.1080/13880209.2017.1314511

**Published:** 2017-04-14

**Authors:** Gökçe Şeker Karatoprak, Fatih Göger, Mükerrem Betül Yerer, Müberra Koşar

**Affiliations:** a Department of Pharmacognosy, Pharmacology, Faculty of Pharmacy, Erciyes University, Kayseri, Turkey;; b Department of Pharmacognosy, Faculty of Pharmacy, Anadolu University, Eskişehir, Turkey;; c Department of Pharmacognosy, Faculty of Pharmacy, Eastern Mediterranean University, Gazimağusa, Mersin, Turkey

**Keywords:** Geraniaceae, antioxidant activity, HPLC/MS/MS, A549 cells

## Abstract

**Context:** *Pelargonium endlicherianum* Fenzl. (Geraniaceae) roots and flowers are traditionally used in Turkey as a decoction treatment against intestinal parasites. Neither the chemical composition nor the potential bioactivity of the plant roots has been studied before.

**Objectives:** The phenolic content and effects of *P. endlicherianum* root extracts on antioxidant enzyme levels on A549 cells were studied for the first time.

**Materials and methods:** The chemical composition was analyzed via spectrophotometric and chromatographic (HPLC MS/MS and HPLC) techniques. The antioxidant activity was determined at different concentrations ranging from 0.001 to 2 mg/mL using DPPH^•^ and ABTS^•+^ radical scavenging activity, β-carotene-linoleic acid co-oxidation assay, protection of 2-deoxyribose and bovine brain-derived phospholipids against a hydroxyl radical-mediated degradation assay. Glutathione peroxidase and superoxide dismutase activities were also studied as well as the effects of the extracts on nitric oxide levels on IL-1β stimulated A549 cells.

**Results:** The key parameters for the most active ethyl acetate extract included the following: DPPH^•^ IC_50_: 0.23 mg/mL, TEAC/ABTS: 2.17 mmol/L Trolox, reduction: 0.41 mmol/g AsscE, and protection of lipid peroxidation IC_50_: 0.05 mg/mL. Furthermore, the ethyl acetate extract increased the SOD level significantly compared to control group (4.48 U/mL) at concentrations of 100 and 200 μg/mL SOD, 5.50 and 5.67 U/mL, respectively. Apocynin was identified as the major component, and the ethyl acetate fraction was found to be rich in phenolic compounds.

**Discussion and conclusion:** *Pelargonium endlicherianum* root extracts displayed antioxidant activity and increased the antioxidant enzyme levels in IL-1β stimulated A549 cells, while decreasing the NO levels.

## Introduction

*Pelargonium* (Geraniaceae) is a genus of perennial shrubs that includes approximately 270 species, and approximately 80% of the genus is indigenous to South Africa (Baytop 1999). *Pelargonium sidoides* DC. is one geophytic species used as a traditional medicine in South Africa. Meaty bright-red tubers and rhizomes of this plant have been widely used by various cultural groups to treat diarrhea, respiratory tract infections, dysentery and tuberculosis (Kolodziej 2007). After years of clinical research, a drug internationally known as Umkaloabo (taken from the plant’s local name), is now manufactured using the roots of *P. sidoides* (Brendler & van Wyk 2008).

There are two recorded species of *Pelargonium*s (*P. endlicherianum* Fenzl. and *P. quercetorum* Agnew.) known in Turkish flora. *P. endlicherianum* is known by the common name “solucan otu” (tansy) in Turkey; the decoction prepared from its roots and the fresh flowers of the plant is traditionally used to treat intestinal parasites (Baytop 1999). There are no studies of the activity or chemical composition of the roots of this plant. To date, the biological activity of *P. endlicherianum* in the upper respiratory system has not been investigated; it remains unknown whether this effect is similar to *P. sidoides*, which is now used as a trademark agent against upper respiratory system infections.

The current great interest in naturally derived phenolic antioxidants is associated not only with their protective and therapeutic features, relating to the various diseases caused by oxidative damage, but also with their ability to prolong the lifetime of food products (Matkowski et al. 2008). It has been established that free radicals induce oxidative damage in biomolecules (lipids, proteins, nucleic acids) and therefore, cause atherosclerosis, aging, cancer, diabetes, inflammation, AIDS and various degenerative diseases in humans (Halliwell 1994; Pincemail 1995; Dreher & Junod 1996).

A549 cells are non-small adenocarcinomic alveolar basal epithelial cells. These cells are able to synthesize lecithin and contain a high percentage of unsaturated fatty acids, which are utilized by the cytidine-diphosphate-choline pathway and are important for the maintenance of membrane phospholipids in cells. Because the cell membranes are rich in unsaturated fatty acids, these cells are more sensitive to oxidative stress, which can be triggered by inflammatory reactions (Balis et al. 1984). The immune system and reactive oxygen species are correlated in many ways. Inflammatory reactions induce the production of reactive oxygen species (ROS) and vice versa. The effect of antioxidant intake to inflammation due to proinflammatory ROS was extensively investigated. The results revealed that antioxidants may provide a beneficial approach to attenuate the cellular injury and dysfunction observed in some inflammatory disorders (Conner & Grisham 1996; Kelkka 2013).

For all these reasons, we aimed to investigate the activity of *P. endlicherianum* root extracts on the upper respiratory system, to determine whether it has similar effects to *P. sidoides* and to identify the active chemical compounds. The A549 cell line is an alveolar basal cell of the respiratory system that is sensitive to oxidative stress under inflammatory conditions. Inflammation in these cell lines is triggered by a proinflammatory cytokine called IL-1β, and the effects of *P. endlicherianum* have been investigated for endogenous antioxidant enzyme activities and nitric oxide levels. This is the first study to reveal the effects of *P. endlicherianum* on an inflammation model of the upper respiratory system (the IL-1β-induced A549 cell line) in real time.

## Materials and methods

### Plant material and reagents

Plant material was collected from Eskişehir, Dağküplü village in August 2013 and identified by Prof. Müberra Kosar. A voucher specimen was deposited at the herbarium at Anadolu University, Faculty of Pharmacy, Eskişehir, Turkey (ESSE 14453). Chromatographic standards were purchased from the Sigma Chemical Company (St. Louis, MO). All remaining reagents were of the highest purity available and obtained from the Sigma Chemical Company (St. Louis, MO).

### Preparation of the extracts

The dried *P. endlicherianum* root (500 g) was powdered and 250 g of plant material was extracted with 750 mL of 70% methanol. The same amount of plant material was extracted with 750 mL of 11% ethanol for 24 h at 40 °C in a water bath with shaking. This procedure was repeated three times using the same batch of starting material, the resultant filtrates were combined and the solvent was removed under vacuum (40 °C). The 70% Methanol extract (53.44 g) was then fractioned with ethyl acetate and butanol. Both the ethyl acetate and butanol solvent were evaporated to dryness under vacuum. All extracts were lyophilized and stored at −20 °C until analysis.

### Total phenolics, flavonoids, and flavonols

Total phenols were estimated as gallic acid equivalents (GAE) per gram of extract (Singleton et al. 1999). The total flavonoid content was estimated as catechin (CA) equivalents using an aluminum chloride colorimetric assay (Zhishen et al. 1999). Total flavonols were estimated as rutin equivalents (RE) and expressed as mg rutin eq/g extract (Miliauskas et al. 2004). All determinations were performed in triplicate and the mean values were calculated.

### Qualitative and quantitative chromatographic analysis with LC-MS/MS and HPLC systems

Experiments were performed using a Shimadzu 20 A HPLC system coupled to an Applied Biosystems 3200 Q-Trap LC-MS/MS instrument equipped with an ESI ion source and used in the negative ionization mode. Separations were performed on an ODS 150 × 4.6 mm, i.d. 3 μm, octadecyl silica gel analytical column operating at 40 °C and a flow rate of 1 mL/min. Elution was performed using a binary gradient of methanol/water/formic acid (10/89/1, v/v/v) (solvent A) and methanol/water/formic acid (89/10/1, v/v/v) (solvent B). The composition of B was increased from 15% to 40% in 15 min, increased to 45% in 3 min and held for 17 min and increased to 100% in 10 min; the composition was then decreased to 15% in 5 min.

The HPLC experiments were performed using an Agilent HP 1100 HPLC-diode array detection (DAD) system equipped with an autosampler (Agilent, Waldbronn, Germany). Separations were performed on a 250 × 4.6 mm i.d., 5 μm reverse-phase Mediterrenean-C18 analytical column operating at room temperature (22 °C) at a flow rate of 1 mL/min. Detection was from 200 to 550 nm. Elution was carried out using a ternary non-linear gradient of a solvent mixture of methanol/water/acetic acid (10:88:2, v/v/v) (solvent A), methanol/water/acetic acid (90:8:2, v/v/v) (solvent B) and methanol (solvent C). The composition of B was increased from 15% to 30% in 15 min, increased to 40% in 3 min and held for 12 min, increased to 100% in 5 min; then, the composition of C was increased to 15% in 2 min, increased to 30% in 11 min and returned to the initial conditions in 2 min. A 10-min equilibrium time was allowed between injections. All standard and sample solutions were injected in triplicate.

### Iron (III) to iron (II) reduction activity

The ability of the extracts to reduce iron (III) was assessed using the method of Oyaizu (1986). The reductive activities of the extracts were expressed as ascorbic acid equivalents (AscAE) in mmol ascorbic acid/g sample (Kosar et al. 2008). Larger AscAE values indicate greater reducing power. The data are presented as the mean value of triplicate analyses.

### 1,1-Diphenyl-2-picrylhydrazyl radical (DPPH^•^) scavenging activity

The ability of the extracts to scavenge DPPH^•^ was determined using the method of Gyamfi et al. (1999). The % inhibition was calculated using Equation (1). Estimated IC_50_ values are presented as the mean value of triplicate analyses.

(1) $$\%\text{ inhibition}=\left[\left({\text{Abs}}_{\text{control}}–{\text{ Abs}}_{\text{sample}}\right)/{\text{Abs}}_{\text{control}}\right]\times \text{1}00$$

### Determination of inhibition of β-carotene/linoleic acid co-oxidation

The antioxidant activity of extracts of *P. endlicherianum* was determined according to the β-carotene bleaching method (Oomah & Mazza 1996). Absorbance values were measured using a spectrophotometer at 470 nm. According to the literature, samples were then subjected to thermal autoxidation in a constant-temperature water bath at 50 °C for 2 h to accelerate oxidation. The rate of β-carotene bleaching was monitored by taking the absorbance at 15 min intervals. Antioxidative activity was calculated according to Oomah and Mazza (1996) (Equation (2)).

(2) $$\text{AAC}=\;\left[\left({\text{Ab}}^{\text{12}0}{}_{\text{sample}}-{\text{ Abs}}^{\text{12}0}{}_{\text{control}}\right)/\left({\text{Ab}}^{0}{}_{\text{control}}-{\text{Abs}}^{\text{12}0}{}_{\text{control}}\right)\right]\times \text{1}00$$

### 2,2-Azinobis(3-ethylbenzothiazoline-6-sulfonate) radical (ABTS^•+^) scavenging activity

To further confirm the free radical scavenging activity of the extracts, an alternative synthetic 2,2-azinobis(3-ethylbenzothiazoline-6-sulfonate) radical (ABTS^•+^) model was used. Absorbance was measured on a UV/spectrophotometer at 734 nm (Papandreou et al. 2006).

### Ascorbate-iron (III)-catalyzed phospholipid peroxidation

The ability of the extracts to scavenge hydroxyl radicals was determined using the method of Aruoma et al. (1997). The % inhibition was calculated using Equation (1). Estimated IC_50_ values are presented as the mean value of triplicate analyses.

### Non-site specific hydroxyl radical mediated 2-deoxy-d-ribose degradation

The ability of the extracts to inhibit non-site-specific hydroxyl radical-mediated peroxidation was performed essentially as described by Halliwell et al. (1987). The % inhibition was calculated using Equation (1). Estimated IC_50_ values are presented as the mean value of triplicate analyses.

### Cell culture

Human non-small adenocarcinomic alveolar basal epithelial cells (A549) were grown in 90% RPMI 1640 (Invitrogen, Carlsbad, CA) medium with 1% penicillin, streptomycin mix solution (Invitrogen) and 10% FBS. Cultures were maintained at 37 °C in 5% CO_2_ and 95% air.

### Cell viability

The cell proliferation was studied by using sulforhodamine B (SRB) assay and xCELLigence system^®^, a real time cell analyzer.

### The sulforhodamine B (SRB) colorimetric assay

Cells were inoculated into 96-well microtiter plates (2.5 × 10^4^ in 100 μL). After cell inoculation, the plates were incubated for 24 h prior to the addition of extracts. After extract addition at a final concentration of 125, 250 or 500 μg/mL, plates were incubated at standard conditions for 24 h and cells were fixed with 40% (w/v) TCA and incubated for 1 h at 4 °C. The plates were washed five times with water and air-dried. A 0.4% (w/v) SRB in 1% acetic acid was added and the staining was performed for 30 min at room temperature. The SRB solution was removed by washing the plates quickly with water and then with 15% v/v acetic acid. The bound SRB was solubilized by adding 100 μL of 10 mM unbuffered Tris-Base, and the absorbance was measured at 540 nm (Houghton et al. 2007). The values are represented as the mean values of four measurements.

### Real-time cell analyzer (RTCA) xCELLigence

The xCELLigence system was used according to the instructions provided by the supplier (Acea Biosciences, San Diego, CA 92121). This system provides constant quantitative real-time monitoring of cells based on impedance measurements to analyze the status of the adherent cells *in vitro*.

Cells were inoculated into 96-well e-plates (6.25 × 10^3^ in 100 μL). Twenty-four hours later when the cell index was approximately 1 (in the linear log growth phase), extracts were added at a final concentration ranging between 125 and 500 μg/mL. Cells growing in the culture medium were included as untreated cells or negative control. The e-plate was placed into the cell culture incubator. The adhesion growth and proliferation of the cells were monitored for up to 48 h via the incorporated sensor electrode arrays of the e-plate. The electrical impedance was measured using the RTCA-integrated software of the xCELLigence system as a dimensionless parameter termed Cell Index (CI).

After investigating the cytotoxicity of extracts using an SRB cytotoxicity assay and xCELLigence cell analyzer, a new study was designed to investigate the effect of lower concentrations of extracts and IL-1β treatment on A549 cells.

### Treatment of extracts with IL-1β

In the second study using the xCELLigence system, 6.25 × 10^3^ cells in 100 μL were inoculated into 96-well e-plates. After the cell index reached 1 (linear log growth phase), 2% FBS and 1 ng/mL IL-1β containing medium were added to obtain the IL-1β (−) and IL-1β (+) groups, respectively. Six hours later, 2% FBS containing medium was added to the IL-1β (−) group and 5 ng/mL concentrated IL-1β was added to the IL-1β (+) groups to ensure the development of inflammation. After 3 h incubation, the 11% ethanol, 70% methanol, ethyl acetate and butanol extracts were added to the wells at 50, 100 and 200 μg/mL final concentrations. Cells growing in the culture medium were included as untreated cells or negative control. The e-plate was placed into the cell culture incubator and monitored for 48 h.

### Glutathione peroxidase (GSH-Px) and superoxide dismutase (SOD) enzymes activities

To detect GSH-Px and SOD activity, cells were seeded to 1 × 10^6^ and 24 h after 1 ng/mL concentrated IL-1β was added to the IL-1β (+) groups. After 3 h, extracts were added to the wells at 50, 100 and 200 μg/mL final concentrations. Cells growing in the culture medium were also included as untreated cells or as a negative control. Plates were incubated for 3 h. The supernatant of the cells in each well was centrifuged for 15 min at 10,000 *g* at 4 °C to remove cellular debris. Cytosolic GSH-Px (Biovision, Mountain View, CA) and SOD activities (Cayman, Ann Harbor, MI) were measured using commercial kits according to the kit procedure. 

### Measurement of NO level

Each 50 μL of culture supernatant was mixed with an equal volume of Griess reagent (0.1% *N*-(1-naphthyl)-ethylenediamine, 1% sulfanilamide in 5% phosphoric acid) and incubated at room temperature for 10 min. The absorbance at 550 nm was measured in a multifunctional microplate reader (Bio-Tek, Synergy HT) and a series of known concentrations of sodium nitrite were used as standards.

### Statistical analysis

Data are presented as the mean values ± 95% confidence interval. Analysis of variance was performed using ANOVA procedures. Significant differences between means were determined by Tukey’s pairwise comparison test at a level of *p* < 0.05.

## Results

### Total phenolics, flavonoids and flavonols

The total phenol and flavonoid content of the plant was calculated spectrophotometrically. The highest content was found in the ethyl acetate extract (305.91 ± 10.52 mg_GAE_/g_extract_, 82.16 ± 0.68 mg_CA_/g_extract_, respectively). The extract with the highest total flavonol content was the 70% methanol extract (34.75 ± 1.61 mg_RE_/g_extract_) (Table 1).

**Table 1.** Extract yields, total phenols, flavonoids and flavonols data for *Pelargonium endlicherianum*.

		Spectrophotometric results^b^		
Sample	Yield^a^ [%]	Total phenols^c^	Total flavonoids^d^	Total flavonols^e^
(Pe 11)	%7.57	173.93 ± 7.72	36.03 ± 0.76	16.26 ± 1.47
(Pe 70)	%21.37	201.85 ± 6.44	41.70 ± 0.46	34.75 ± 1.61
(Pe etac)	%16.60	305.91 ± 10.52	82.16 ± 0.68	26.64 ± 1.68
(Pe but)	%20.50	139.61 ± 1.14	27.50 ± 0.52	9.25 ± 1.80

(Pe 11), 11% ethanol extract; (Pe 70), 70% methanol extract; (Pe etac), ethyl acetate extract; (Pe but), *n*-butanol extract.

a Extract yields expressed as milligrams of extract per gram (dry weight) of material.

b Values (mg/g) are expressed as means** **±** **standard error.

c Total phenols expressed as gallic acid equivalents: milligrams of gallic acid per gram (dry weight) of extract.

d Total flavonoids expressed as catechin equivalents: milligrams of catechin per gram (dry weight) of extract.

e Total flavonols expressed as rutin equivalents: milligrams of rutin per gram (dry weight) of extract.

### Qualitative–quantitative chromatographic analysis by LC-MS/MS and HPLC

**Figure 1.**
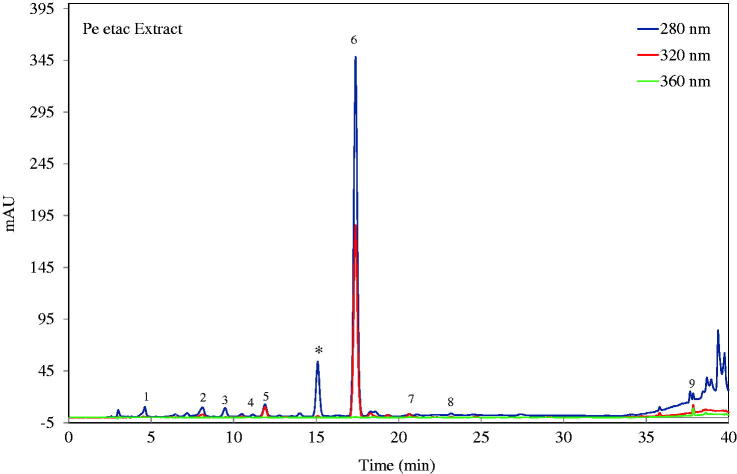
HPLC-PDA chromatogram of ethyl acetate extract of *P. endlicherianum*. (numbers are in Table 3; * is unidentified).

The chemical compositions of the extracts were determined by LC/MS/MS and HPLC analyses. Standards were determined according to mass analyses and quantitative analyses using reversed-phase chromatography. Apocynin (1-(4-hydroxy-3-methoxyphenyl) ethanone) was identified in the ethyl acetate, 70% methanol and 11% ethanol extracts and was the major compound in all these extracts. Additionally, benzoic acid derivatives (gallic acid, 3,5-dihydroxybenzoic acid and vanillic acid), hydroxycinnamic acid derivatives (ferulic acid and caffeic acid), the flavonoid derivative quercetin and the tannin derivative 1,2,3,4,6 pentagalloyl glucose (PGG) were detected in the extracts (Tables 2 and 3). An HPLC chromatogram of the ethyl acetate extract is shown in Figure 1.

**Table 2.** LC/MS/MS results of *Pelargonium endlicherianum*.

Identified components	Rt	Mw [M-H]^-^	Fragment ions (*m*/*z*)	Extracts^a^
Gallic acid	4.50	169	125, 97	Pe 11, Pe 70, Pe etac, Pe but
Dihydroxy benzoic acid	7	153	109	Pe etac Pe 11
Caffeic acid	11.40	179	135	Pe etac
Homovanillic acid	10.90	181	166,138, 109	Pe etac Pe 70
Apocynin	15.50	165	150, 122	Pe 70 Pe etac Pe 11
1,2,3,4,6-Pentagalloyl glucose	15.04	939	469 (M-2H)^2-,^ 617, 769, 393, 317, 241, 211, 169, 125	Pe 11, Pe 70, Pe etac

a (Pe 11), 11% ethanol extract; (Pe 70), 70% methanol extract; (Pe etac), ethyl acetate extract; (Pe but), *n*-butanol extract.

**Table 3.** Qualitative and quantitative HPLC data for *Pelargonium endlicherianum*.

	Extracts^a^			
Identified components	(Pe 11)	(Pe 70)	(Pe etac)	(Pe but)
1- Gallic acid	1.070 ± 0.004^b^	0.458 ± 0.008	0.968 ± 0.004	0.150 ± 0.000
2-3,5-Dihydroxy benzoic acid	n.d.	n.d.	3.199 ± 0.052	n.d.
3-Caffeic acid	n.d.	n.d.	0.072 ± 0.003	n.d.
4-Homovanillic acid	t.a	t.a	t.a	n.d.
5-Vanillic acid	t.a	t.a	t.a	n.d.
6-Apocynin	2.486 ± 0.002	3.509 ± 0.016	17.378 ± 0.053	0.592 ± 0.000
7-1,2,3,4,6-Pentagalloyl glucose	n.d.	n.d.	0.541 ± 0.001	n.d.
8-Ferulic acid	n.d.	n.d.	0.084 ± 0.000	n.d.
9-Quercetin	n.d.	0.554 ± 0.008	0.547 ± 0.001	n.d.

a (Pe 11), 11% ethanol extract; (Pe 70), 70% methanol extract; (Pe etac), ethyl acetate extract; (Pe but), *n*-butanol extract.

b % Extract: means** **±** **standard error; n.d.: not detected; t.a.: trace amount.

### Iron (III) to iron (II) reduction activity

The ability of all the extracts to reduce ferric iron to ferrous iron was investigated, and the results are shown in Table 4 as AscAE. According to Table 4, the reducing capacities of the extracts were 0.41–0.81 mmol/g AscAE, and the most active extract was the ethyl acetate extract (0.81 mmol/g AscAE). No extract was as effective as the positive controls ascorbic acid, BHT, BHA, rosmarinic acid and gallic acid. Vanillic acid was found to be less active than the extracts, and apocynin showed no activity even at 10 mg/mL.

**Table 4.** The antioxidant activity results of *Pelargonium endlicherianum* extracts and standards.

Sample^A^	AscAe^B^ (mmol/g extract)	DPPH^C^ IC_50_ (mg/mL)	TBA^D^ IC_50_ (mg/mL)	Non-site specific hydroxyl radical scavenging^E^ IC_50_ (mg/mL)	TEAC^F^ (mmol/L Trolox)
BHT	2.27 ± 0.01^d^	0.08 ± 0.00^b^	0.001 ± 0.00^a^	–	0.62 ± 0.35^+^ 1.29 ± 0.3 ^++^
BHA	2.30 ± 0.00^d^	0.14 ± 0.02^c^	0.001 ± 0.00^a^	–	1.02 ± 0.05^+^ 2.26 ± 0.09^++^
RA	3.75 ± 0.09^c^	0.004 ± 0.0^a^	0.07 ± 0.00^b,c^	–	0.90 ± 0.10^+^ 2.04 ± 0.16^++^
GA	5.58 ± 0.01^a^	0.02 ± 0.00^a^	0.17 ± 0.00^d^	–	2.53 ± 0.13^+^ 2.58 ± 0.02^++^
AscAs	5.37 ± 0.0^b^	0.15 ± 0.01^c^	–	1.27 ± 0.15^b^	1.19 ± 0.05^+^ 2.38 ± 0.02^++^
VA	0.28 ± 0.00^h^	0.2 ± 0.01^d^	5.0 <	5.0 <	1.05 ± 0.07^+++^
Apocynin	10.0 <	10.0 <	3.68 ± 0.09	5.0 <	1.19 ± 0.07^+++^
Pe 11	0.72 ± 0.03^e^	0.63 ± 0.03^f^	0.21 ± 0.01^e^	0.064 ± 0.01^a^	1.09 ± 0.01 ^++^ 1.68 ± 0.14^+++^
Pe 70	0.57 ± 0.02^f^	0.23 ± 0.01^e^	0.1 ± 0.00^c^	0.002 ± 0.00^a^	0.98 ± 0.15^++^ 1.76 ± 0.22^+++^
Pe etac	0.80 ± 0.02^e^	0.23 ± 0.01^e^	0.05 ± 0.00^b^	0.002 ± 0.00^a^	2.02 ± 0.14^++^ 2.17 ± 0.15^+++^
Pe but	0.41 ± 0.00^g^	0.84 ± 0.02^g^	0.72 ± 0.03^f^	0.176 ± 0.02^a^	0.73 ± 0.05^++^ 0.93 ± 0.03^+++^

A BHT: butylated hydroxytoluene; BHA: butylated hydroxyanisole; RA: rosmarinic acid; GA: gallic acid; AscAs: ascorbic acid; VA: vanillic acid; Apocynin: apocynin; (Pe 11): 11% Ethanol extract; (Pe 70): 70% Methanol extract; (Pe etac): Ethyl acetate extract; (Pe but): *n*-Butanol extract.

B Iron (III) reduction.

C DPPH radical scavenging.

D Inhibiton of malondialdehyde formation.

E Non-site specific hydroxyl radical scavenging.

F TEAC is defined as the concentration of Trolox (mmol/L) having the ABTS**^•^**^+^ scavenging activity equal to; ^+^ 0,25** **mg/mL, ^++^ 0,5** **mg/mL, ^+++^ 1** **mg/mL sample dilution. Values (mg/mL) expressed as mean** **±** **standard errors (*n*** **=** **4). Bars with the same lower case letters (a–h) are not significantly (*p**** ***>*** ***0.05) different.

### 1,1-Diphenyl-2-picrylhydrazyl radical (DPPH^•^) scavenging activity

The capacities of the extracts to scavenge the DPPH^•^ radical, which is a nitrogen-centered stable radical, were investigated at physiological pH. The IC_50_ values of extracts varied from 0.23 to 0.84 mg/mL (Table 4). The DPPH^•^ radical-scavenging effect registered an IC_50_ of 0.23 mg/mL, and the 70% methanol and ethyl acetate extracts were found to be more active than the other extracts. The DPPH^•^ radical-scavenging effect of apocynin, which is the main compound in the extracts, was indicated by an IC_50_ level greater than 10 mg/mL; the scavenging activity of the extracts did not appear to result from apocynin. The IC_50_ value of vanillic acid was 0.199 mg/mL and was more active than the ethyl acetate and 70% methanol extracts. The IC_50_ value of the gallic acid, which was used as a positive control and was also a component of the extracts, was detected to be 0.023 mg/mL. Its radical-scavenging effect was found to be higher than that of BHA, BHT and ascorbic acid.

### Determination of inhibition of β-carotene/linoleic acid co-oxidation

**Figure 2.**
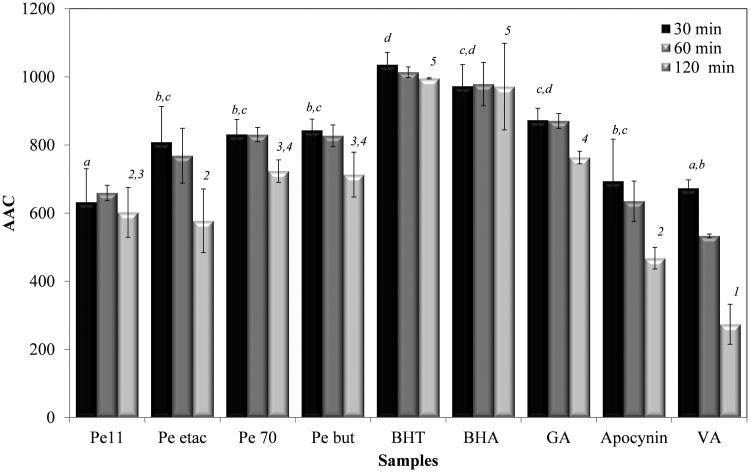
The effect of the extracts and positive controls on β-carotene/linoleic acid co-oxidation (Pe 11), 11% ethanol extract; (Pe 70), 70% methanol extract; (Pe etac), ethyl acetate extract; (Pe but), *n*-butanol extract; bars with the same lower case letter and number (a–d), (1–5) are not significantly (*p**** ***>*** ***0.05) different.

The oxidation-inhibiting effect of extracts was investigated in a time-dependent manner, and time-dependent alteration was observed. The inhibition percentages of all extracts are given in Figure 2. According to Figure 2, all extracts inhibited oxidation. The 70% methanol, butanol and ethyl acetate extracts displayed statistically similar activities (*p* > 0.05) after 30 min and were more active than the 11% ethanol extract. All extracts showed less activity than the positive controls BHT and BHA. Gallic acid was found to inhibit oxidation to a statistically equivalent degree (*p* > 0.05) as the 70% methanol, butanol and ethyl acetate extract, while apocynin and vanillic acid were less active than these extracts.

### ABTS^•+^ radical scavenging

In this study, the ABTS^•+^ radical-scavenging effects of the extracts and vanillic acid were studied at concentrations of 0.50 and 1 mg/mL. The standards concentrations were 0.25 and 0.5 mg/mL, while apocynin was studied at 1 and 5 mg/mL. All extracts revealed the highest activity at 1 mg/mL. No extract surpassed the activity of BHT, BHA, gallic acid and rosmarinic acid, which were used as positive controls (Table 4). The TEAC value of the ethyl acetate extract was determined to be 2.17 mmol/L Trolox at a 1 mg/mL concentration and was found to be more active than BHT. The hierarchy of activity of the extracts for ABTS^•+^ radical-scavenging was ethyl acetate > 70% methanol > 11% ethanol > butanol. The activity of vanillic acid and apocynin at 1 mg/mL concentration (TEAC: 1.05 and 1.19 mmol/L Trolox, respectively) were found less active than the ethyl acetate, 70% methanol, 11% ethanol extracts.

### Ascorbate-iron (III)-catalyzed phospholipid peroxidation

In this study, phospholipid-based liposomes in the presence of iron (III) and ascorbic acid were quickly subjected to hydroxyl radical-induced peroxidation, which gave malondialdehyde and similar aldehydes. The extracts showed strong antioxidant effects by inhibiting the formation of 2-thiobarbituric acid-reactive substances (TBARS). Among the extracts, the ethyl acetate extract, which is rich in tannins and phenolic acids, was determined to be most active (IC_50_: 0.05 mg/mL). The butanol extract had the highest IC_50_ value (0.72 mg/mL) among all extracts. The IC_50_ values of apocynin and vanillic acid were found to be quite high compared to the extracts (Table 4). None of the extracts were found to be as active as BHT and BHA but the ethyl acetate and 70% methanol extracts were found to be more active than gallic acid (IC_50_: 0.17 mg/mL) (*p* < 0.05).

### Non-site specific hydroxyl radical mediated 2-deoxy-d-ribose degradation

Extracts inhibited nonspecific hydroxyl radical-mediated 2-deoxyribose degradation more strongly than did ascorbic acid. All extracts displayed statistically similar activities (*p* > 0.05), with IC_50_ values ranging from 0.002 to 0.176 mg/mL (Table 4). Additionally, the lipid peroxidation-inhibiting capacities and nonspecific hydroxyl radical-mediated 2-deoxyribose degradation-inhibiting effects of the extracts were found to be highly correlated (*y* = 3.73*x* + 0.43; *R*^2^ = 0.975).

### GSH-Px and SOD activities

**Figure 3.**
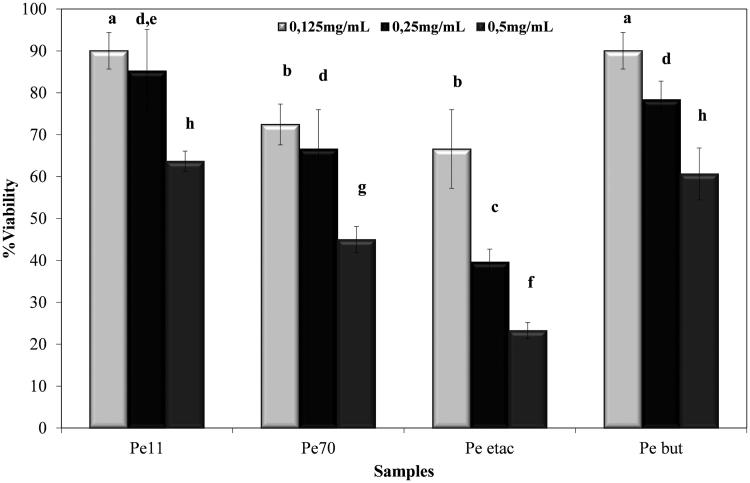
Cytotoxicity effects of the extracts of *P. endlicherianum* extracts. (Pe 11), 11% Ethanol extract; (Pe 70), 70% methanol extract; (Pe etac), ethyl acetate extract; (Pe but), *n*-butanol extract; bars with the same lower case letter (a–h) are not significantly (*p**** ***>*** ***0.05) different.

**Figure 4.**
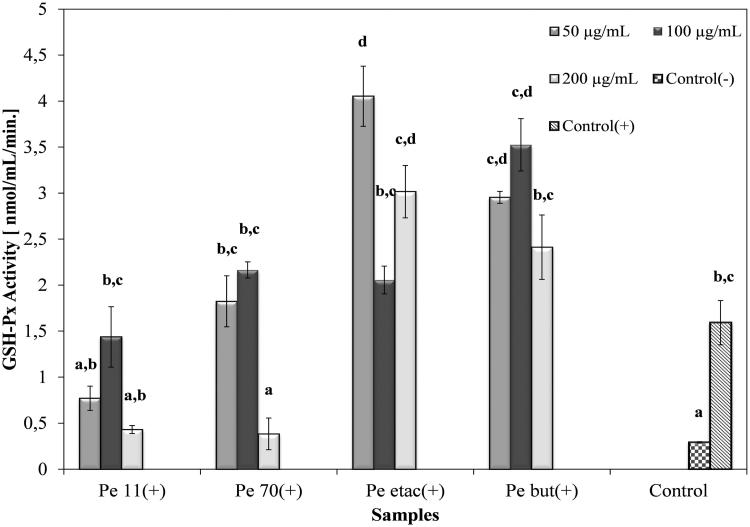
The GSH-Px activity of *P. endlicherianum* extracts on IL-1β treated A549 cells. (Pe 11), 11% Ethanol extract; (Pe 70), 70% methanol extract; (Pe etac), ethyl acetate extract; (Pe but), *n*-butanol extract; bars with the same lower case letter (a–d) are not significantly (*p**** ***>*** ***0.05) different.

**Figure 5.**
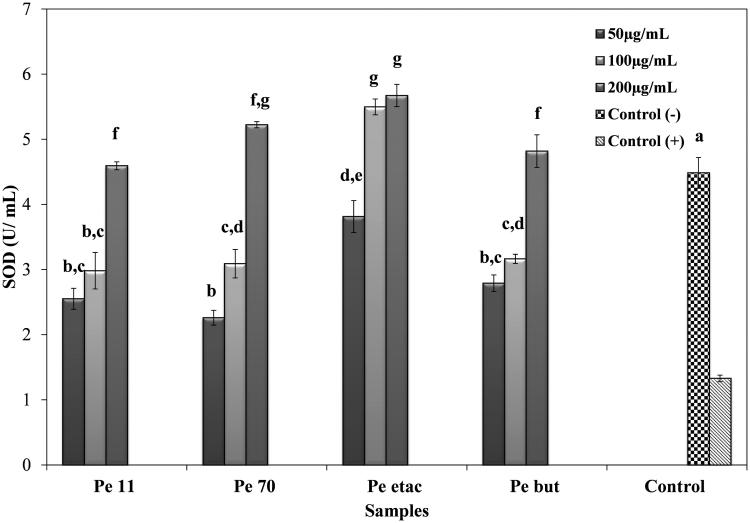
The SOD activity of *P. endlicherianum* extracts on IL-1β treated A549 cells. (Pe 11), 11% Ethanol extract; (Pe 70), 70% methanol extract; (Pe etac), ethyl acetate extract; (Pe but), *n*-butanol extract; bars with the same lower case letter (a–g) are not significantly (*p**** ***>*** ***0.05) different.

In cytotoxicity studies for 250 and 500 μg/mL concentrated extracts, the viability of the A549 cell line decreased for both concentrations according to the SRB assay (Figure 3) and RTCA. In the second study with lower concentrations, 24 h cell index data were collected for RTCA (Tables 5 and 6). Compared to the control, extracts were not found to be cytotoxic at 50, 100 and 200 μg/mL concentrations. Further activities of the extracts related to the *in vitro* antioxidant capacity of the GSH-Px and SOD enzyme activities were investigated at the same concentrations (Figures 4 and 5). Endogenous antioxidant enzyme levels were investigated with the development of inflammation. Following incubation of A549 cells with IL-1β, GSH-Px levels increased significantly (*p* < 0.05) compared to the IL-1β (−) group. In the IL-1β (+) group, the ethyl acetate extract and butanol extract increased GSH-Px activity compared to the control at the 50 μg/mL concentration. The most significant increase (*p* < 0.05) was observed in the 50 μg/mL ethyl acetate extract, at 4.05 mmol/mL/min. The 70% methanol extract and 11% ethanol extract reduced the activity of GSH-Px more than the control at 200 μg/mL, at 0.43 and 0.38 mmol/mL/min, respectively. The butanol extract did not reduce the activity of GSH-Px compared to the control at any concentration and showed no significant difference between the concentrations (*p >* 0.05) (Figure 4).

**Table 5.** The 24** **h cell index data of the extracts and control on A549 cells in IL-1β (−) group.

**Control:** 2.52855			
Extracts^a^	50 μg/mL	100 μg/mL	200 μg/mL
Pe 11	2.7707	2.6914	2.4244
Pe 70	2.8794	2.9351	3.3015
Pe etac	2.3759	2.9310	2.7858
Pe but	2.2624	2.6345	3.0275

a (Pe 11): 11% Ethanol extract; (Pe 70): 70% Methanol extract; (Pe etac): Ethyl acetate extract; (Pe but): *n*-Butanol extract.

**Table 6.** The 24** **h cell index data of the extracts and control on A549 cells in IL-1β (+) group.

**Control:** 2.72588			
Extracts	50 μg/mL	100 μg/mL	200 μg/mL
Pe 11	3.4804	3.3382	2.7441
Pe 70	2.4184	3.4060	3.0060
Pe etac	2.7291	2.6210	2.8363
Pe but	2.8600	2.3949	2.5758

(Pe 11): 11% Ethanol extract; (Pe 70): 70% Methanol extract; (Pe etac): Ethyl acetate extract; (Pe but): n-Butanol extract.

SOD activity in A549 cells was assessed by measuring cytosolic Cu/Zn SOD levels. Following incubation of A549 cells with IL-1β, SOD levels decreased significantly in the IL-1β (+) group compared to the IL-1β (−) group. In the IL-1β (+) group, extracts increased SOD levels in a dose-dependent manner. According to Figure 5, the ethyl acetate extract increased SOD levels most significantly (*p* < 0.05) compared to the IL-1β (+) control group at the 100 and 200 μg/mL concentrations. The SOD levels were 5.50 U/mL and 5.67 U/mL, respectively.

### Measurement of NO levels

**Figure 6.**
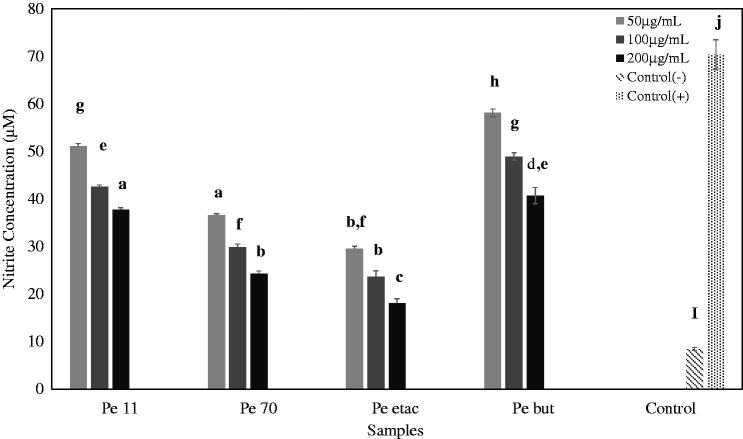
The effects of the *P. endlicherianum* extracts on NO inhibition in IL-1β treated A549 cells. 11% Ethanol extract; (Pe 70), 70% methanol extract; (Pe etac), ethyl acetate extract; (Pe but), *n*-butanol extract; bars with the same lower case letter and number (a–j) are not significantly (*p**** ***>*** ***0.05) different.

The effects of *P. endlicherianum* extracts on NO inhibition were determined after treating A549 cells with IL-1β. NO levels increased significantly (*p* < 0.05) in the IL-1β (+) control group compared to the control IL-1β (−) group. According to Figure 6, the extracts decreased in NO level in a dose-dependent manner. In particular, the ethyl acetate extract decreased significantly in NO level (*p* < 0.05) at all the concentrations compared to the IL-1β (+) control group. At 50, 100 and 200 μg/mL concentrations, nitrite levels were 29.56 μM, 23.56 μM, 18.1 μM, respectively.

## Discussion

The chemical composition and biological activity of *P. endlicherianum* have not yet been identified; the results of our study are the known first record of this plant, its chemical composition and its biological activity. According to our results, apocynin was identified as the main compound in the extracts. Apocynin is also known as acetovanillone; it is a derivative of vanillin and vanillic acid, which is itself the oxidized form of vanillin. A possible synthesis pathway for apocynin in plants is through the 4-hydroxybenzoic acid derivatives vanillin and vanillic acid. These 4-hydroxybenzoic acid derivatives are produced in the shikimic acid pathway either directly or through cleavage of the double bonds of cinnamic acid derivatives and the reduction of two carbons from the adjacent chain. Among these, *p*-coumaric acid is the precursor of 4-hydroxybenzoic acid and ferulic acid in the production of vanillic acid (Dewick 2002). The presence of benzoic acid derivatives and ferulic acid in the extracts suggests that these molecules are precursors of apocynin. In the literature, it is reported that *P. sidoides* and *P. reniforme* contain various phenolic and polyphenolic compounds and that their roots contain 6,7,8-trihydroxy-coumarin and 8-hydroxy-5,6,7-trimethoxy-coumarin. Umckalin, which is structurally associated with coumarin, has been determined to be the parent compound (Saraswathi et al. 2011). In the species *P. inquinans*, the active compound was determined to be 1,2,3,4,6-penta-*O*-galloyl*-*β-d-glucose (Piao et al. 2008). Compositional analyses within the scope of this study did not identify any coumarin derivative compounds in the roots of *P. endlicherianum*.

To identify the biological activities of the *P. endlicherianum* root extracts, the antioxidant capacity of the plant was determined. Different studies showed that the antioxidant activity is related to the development of reductones. Reductones are terminators of free radical chain reactions; thus, the antioxidant activity of the extracts may be related to their reductive activity. Reducing capacity is highly important in the beginning phases of radical chain reactions (Dorman et al. 2003). This method is suitable for the detection of hydrophilic and lipophilic antioxidants (Prior et al. 2005). The chemical composition of the tannin-rich ethyl acetate extract was determined to be the highest electron-donating extract. It was shown that the ability of tannins to reduce iron (III) increased with concentration (Zhang & Lin 2009). Studies found that the iron-reducing activity of PGG (which is more concentrated in the ethyl acetate extract than in other extracts) was higher than ascorbic acid (IC_50_: 48 ± 0.5 μM) with a value of IC_50_: 4.6 ± 0.2 μM (Sancheti et al. 2010).

The compositions of the 70% methanol and ethyl acetate extracts that show the highest level of DPPH^•^ radical scavenging activity are rich in tannin derivatives. These compounds appear to be responsible for the antioxidant effect of *P. endlicherianum* root extracts. PGG, which is present in the ethyl acetate extract, is the pentahydroxy gallic acid ester of glucose and a common precursor of gallotannins and ellagitannins. Previous studies have shown the IC_50_ value of PGG (1.14 μg/mL), as calculated from its DPPH^•^ radical-scavenging activity, and suggested that the compound was a potent antioxidant (Piao et al. 2008).

Lipids in the cell membranes are rich in unsaturated fatty acids that are highly prone to oxidation, such as linoleic acid and arachidonic acid (Braca et al. 2003; Liyana-Pathirana & Shahidi 2006). Thus, it is important to use the assay media from the oxidation of lipids when scanning and investigating the antioxidant effects of the extracts. One of the most common experimental models used for this purpose is the β-carotene-linoleic acid bleaching assay. In this assay, the ethyl acetate, 70% methanol and butanol extracts showed similar activity but no extracts were as active as BHT and BHA. An 11% ethanol extract showed less activity than the other extracts because nonpolar antioxidants are concentrated at the lipid-air interface and demonstrate high protection in emulsions against polar antioxidants present in the aqueous phase (Pizzale et al. 2002).

One alternative synthetic radical is ABTS^•+^, a moderately stable nitrogen-centered radical species. Although the principle underpinning the use of ABTS^•+^ and DPPH^•^ free radicals is essentially identical, ABTS^•+^-based models are more useful for nonpolar and polar samples; additionally, spectral interference is minimized because the absorption maximum used is 760 nm, a wavelength not normally encountered with natural products (Re et al. 1999). From the TEAC data, the concentration of Trolox (mmol/L) with an ABTS^•+^-scavenging activity equal to different concentrations of standards and extracts is as shown in Table 4. The calculated TEAC values for extracts are less than the TEAC values for ascorbic acid, rosmarinic acid, gallic acid, BHT and BHA. Phenolic acids and tannin-rich extracts showed high levels of activity in this trial and were in accordance with the literature. However, vanillic acid showed similar activity to the 11% ethanol, 70% methanol and ethyl acetate extracts at a concentration of 0.5 mg/mL. Apocynin was studied at higher concentrations and was found to be less active than vanillic acid and the extracts.

Lipid peroxidation is the accumulated result of reactive oxygen species and a chain reaction that causes the dysfunction of biological systems (Badmus et al. 2011). Hydroxycinnamic acid derivatives and flavonoids in their free form are dissolved in the lipoid medium. It is well known that lipophilic compounds are more active than hydrophilic compounds in assays of lipid peroxidation (Braca et al. 2003). All extracts demonstrated the ability to inhibit the formation of thiobarbituric acid-reactive substances (TBARS) by scavenging hydroxyl radicals that were generated by ascorbate/iron (III)-dependent Fenton chemistry in a concentration-dependent manner. Based on these results, the high levels of activity in the ethyl acetate and 70% methanol extracts are associated with a rich phenolic composition, especially in the presence of PGG.

Hydroxyl radicals are one of the most reactive free radicals that damage biological molecules in living organisms. Through mediation of nonspecific (Fe^2+^+H_2_O_2_+EDTA) and specific (Fe^2+^+H_2_O_2_) hydroxyl radicals in the *in vitro* Fenton’s reaction system, the OH^•^ radical attacks deoxyribose and converts it to TBARS. Phenolic groups in the extracts are responsible for OH^•^ radical-scavenging. The 70% methanol and ethyl acetate extracts, which showed the highest levels of OH^•^ radical-scavenging activity, are rich in phenolic compounds. Thus, the extracts contain compounds that are capable of protecting carbohydrate and lipid components in foods, cosmetics and pharmaceutical preparations from oxidative damage, as mediated through hydroxyl radicals.

ROS generated during inflammation are believed to play critical roles in various diseases (Halliwell 1994; Pincemail 1995; Dreher & Junod 1996). In the literature, it is reported that IL-1β induces reactive oxygen species in A549 cells (Lin et al. 2009; Chen et al. 2000). The enzymatic antioxidant system is principally composed of superoxide dismutases (SODs), catalase (CAT) and glutathione peroxidase (GPX) and exists in the body to prevent ROS toxicity. SOD catalyzes the dismutation of O_2_^•^, resulting in the production of H_2_O_2,_ and also plays a critical but limited role in detoxifying ROS. H_2_O_2_ can cause oxidative stress when H_2_O_2_ detoxification enzymes are ineffective or depleted. CAT and GPX are the principal H_2_O_2_ detoxifying enzymes (Mathy-Hartert et al. 2008). Following exposure of cells to 1 ng/mL IL-1β, the highest GSH-Px level was observed for the 50 μg/mL concentrated ethyl acetate extract *of P. endlicherianum*. These results revealed that *P. endlicherianum* extracts have a protective effect on IL-1β-induced cell damage.

Cytosolic Cu/Zn SOD levels in the control in the IL-1β treated group were significantly lower than the control in the IL-1β non-treated group. Low Cu/Zn SOD in the group that developed inflammation was in accordance with the results of Kruidenier et al. (2003). In their study, it was reported that TNF-α and IL-1β, which increased with inflammation, more rapidly triggered an increase in Mn-SOD but did not affect Cu/Zn SOD levels (Kruidenier et al. 2003). In the IL-1β-treated group, the extracts increased Cu/Zn SOD levels under increased concentrations and showed dose-dependent antioxidant activity.

Epidemiological studies have shown a positive correlation between the consumption of plant foods, which are rich sources of antioxidants, and a reduction in disease risk as mediated by reactive oxygen species. Previous studies have also suggested that plant secondary metabolites act as excellent anti-inflammatory agents and play an important role in oxidative stress and inflammation (Joo et al. 2014). Nitric oxide is an essential bioregulatory molecule required for several physiological processes including neural signal transmission, immune response and control of blood pressure by vasodilatation. However, excessive amounts of NO are associated with a range of inflammatory diseases (Abd El-Wahab et al. 2013). The results of the NO determination demonstrated that IL-1β was found to significantly induce NO production in A549 cells. All extracts, particularly ethyl acetate, significantly decreased nitrite accumulation in IL-1β-stimulated A549 cells in a dose-dependent manner.

In this study, we preliminarily revealed the biological activity of *P. endlicherianum* root extracts via *in vitro* antioxidant assays, including cell culture studies using the A549 cell line. We sought to shed light on the mechanism of action of the extracts on inflammation-induced oxidative damage triggered by IL-1β. The activity of iNOS increases during inflammation and also during the respiratory burst of the inflammation response that forms peroxynitrite to attack membrane lipids. As this is another oxidative damage mechanism involved in the inflammation process, we evaluated NO levels. All extracts reduced NO levels, and these first findings related to its protective effects against inflammation-induced oxidative damage indicate that the ethyl acetate root extract, in particular, reveals an antioxidant effect during inflammation in a dose-dependent manner. Furthermore, the extracts supported the antioxidant system by increasing the GSH-Px and SOD activities during inflammation. Both sides of the antioxidant balance supported the system from oxidative damage during inflammation; this *P. endlicherianum* biological activity is reported for the first time in this study.

In addition to its *in vitro* antioxidant effects during inflammation, the effects of *P. endlicherianum* root extracts on A549 cell viability have also been revealed for the first time in this study using the SRB test and a Real Time Cell Analyzer (xCELLigence) system. The root extracts were nontoxic at doses of 50, 100 and 200 μg/mL. These results are the first preliminary findings related to safety studies for *P. endlicherianum* alone or with inflammation. Because the trademark use of *P. sidoides* is in upper respiratory system disorders related to inflammation, we performed a first drug safety study for *P. endlicherianum* root extracts in anticipation of its future clinical use to support preclinical (Phase 0) studies related to the clinical trial potential of the plant.

## Conclusions

Our study indicates for the first time that *P. endlicherianum* extracts play important roles in the modulation of A549 cells in oxidative stress and inflammation and that this modulation of GSH-Px and SOD may represent an important therapeutic target for the treatment of inflammation-related diseases, as well as aging-related diseases in which oxidative stress plays a major role. We observed that the *P. endlicherianum*, 70% methanol extract and ethyl acetate fractions had strong antioxidant activity; these fractions protected against lipid peroxidation and deoxyribose degradation. In summary, the non-polar extracts of *P. endlicherianum* possess antioxidant activities that increase SOD and GSH-Px activity and decrease NO accumulation in A549 cells during inflammation.
